# (*E*,*E*)-1,2-Bis[1-(2-bromo­phen­yl)ethyl­idene]hydrazine

**DOI:** 10.1107/S1600536810029867

**Published:** 2010-07-31

**Authors:** Patcharaporn Jansrisewangwong, Suchada Chantrapromma, Hoong-Kun Fun

**Affiliations:** a Department of Chemistry and Center of Excellence for Innovation in Chemistry, Faculty of Science, Prince of Songkla University, Hat-Yai, Songkhla 90112, Thailand; b Crystal Materials Research Unit, Department of Chemistry, Faculty of Science, Prince of Songkla University, Hat-Yai, Songkhla 90112, Thailand; c X-ray Crystallography Unit, School of Physics, Universiti Sains Malaysia, 11800 USM, Penang, Malaysia

## Abstract

In the title compound, C_16_H_14_Br_2_N_2_, the complete molecule is generated by a crystallographic twofold axis. The dihedral angle between the two benzene rings is 35.28 (8)° and that between the best planes of two ethyl­idinehydrazine N—N=C—Me units is 87.67 (11)°. Each of these N/N/C/C planes makes a dihedral angle of 63.81 (10)° with the adjacent benzene ring. In the crystal, the mol­ecules are arranged into a layer parallel to the *ac* plane through C—H⋯π inter­actions. C⋯Br short contacts [3.4032 (18)–3.5969 (19) Å] are also observed.

## Related literature

For bond-length data, see: Allen *et al.* (1987 ▶). For a related structure, see: Zhao *et al.* (2006 ▶). For background to and the biological activity of hydro­zones, see: Avaji *et al.* (2009 ▶); El-Tabl *et al.* (2008 ▶); Rollas & Küçükgüzel (2007 ▶). For the stability of the temperature controller used in the data collection, see: Cosier & Glazer (1986 ▶).
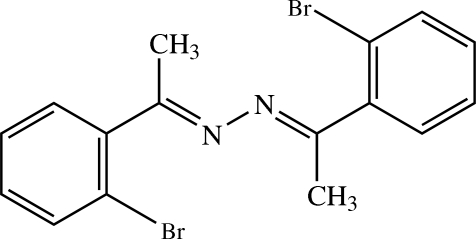


## Experimental

### 

#### Crystal data

C_16_H_14_Br_2_N_2_
*M* *_r_* = 394.11
Orthorhombic, 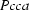

*a* = 17.2162 (3) Å
*b* = 11.8414 (3) Å
*c* = 7.6953 (2) Å
*V* = 1568.79 (6) Å^3^
*Z* = 4
Mo *K*α radiation
μ = 5.16 mm^−1^
*T* = 100 K
0.41 × 0.27 × 0.18 mm

#### Data collection

Bruker APEXII CCD area-detector diffractometer
Absorption correction: multi-scan (*SADABS*; Bruker, 2005 ▶) *T*_min_ = 0.227, *T*_max_ = 0.462
18529 measured reflections
3458 independent reflections
2397 reflections with *I* > 2σ(*I*)
*R*_int_ = 0.042

#### Refinement

*R*[*F* ^2^ > 2σ(*F* ^2^)] = 0.031
*wR*(*F* ^2^) = 0.081
*S* = 1.02
3458 reflections
92 parameters
H-atom parameters constrained
Δρ_max_ = 0.75 e Å^−3^
Δρ_min_ = −0.40 e Å^−3^

### 

Data collection: *APEX2* (Bruker, 2005 ▶); cell refinement: *SAINT* (Bruker, 2005 ▶); data reduction: *SAINT*; program(s) used to solve structure: *SHELXTL* (Sheldrick, 2008 ▶); program(s) used to refine structure: *SHELXTL*; molecular graphics: *SHELXTL*; software used to prepare material for publication: *SHELXTL* and *PLATON* (Spek, 2009 ▶).

## Supplementary Material

Crystal structure: contains datablocks global, I. DOI: 10.1107/S1600536810029867/is2578sup1.cif

Structure factors: contains datablocks I. DOI: 10.1107/S1600536810029867/is2578Isup2.hkl

Additional supplementary materials:  crystallographic information; 3D view; checkCIF report

## Figures and Tables

**Table 1:** Hydrogen-bond geometry (Å, °) *Cg*1 is the centroid of C1–C6 ring.

*D*—H⋯*A*	*D*—H	H⋯*A*	*D*⋯*A*	*D*—H⋯*A*
C3—H3*A*⋯*Cg*1^i^	0.93	2.83	3.7246 (17)	161
C8—H8*A*⋯*Cg*1^ii^	0.96	2.93	3.4989 (18)	119

Symmetry codes: (i) 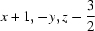; (ii) 
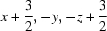
.

**Fig. 1.**
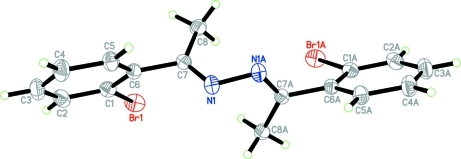
The molecular structure of the title compound, showing 50% probability displacement ellipsoids and the atom-numbering scheme. Atoms with suffix A were generated by symmetry code 1 - x, y, 1/2 - z.

**Fig. 2.**
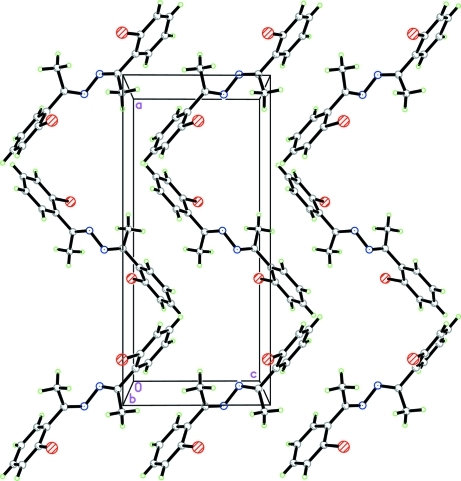
The crystal packing of the title compound viewed along the b axis, showing zigzag chains running along the a-axis.

## supplementary crystallographic information

### Comment

Hydrazones are a special group of compounds in the Schiff base family and characterized by the presence of  >C═N—N═C< (Avaji *et al.*, 2009). They have been studied for their chemical and biological activities for a long time. They and their complexes show various biological activities such as insecticidal, antitumor, antioxidant, antifungal, antibacterial and antiviral properties (El-Tabl *et al.*, 2008; Rollas & Küçükgüzel, 2007). These interesting properties prompt us to synthesise the title hydrazone derivative (I) in order to study its antibacterial activity. Herein the crystal structure of (I) was reported.

The asymmetric unit of (I) (Fig. 1), C_16_H_14_Br_2_N_2_, contains one half-molecule and the complete molecule is generated by a crystallographic symmetry centre 1 - *x*, *y*, 1/2 - *z*. The molecule of (I) exists in an *E* configuration with respect to the C7═N1 double bond [1.2812 (19) Å] and the torsion angle N1A–N1–C7–C6 = -173.12 (13)°. The dihedral angle between the two benzene rings is 35.28 (8)°. Atoms C7/C8/N1/N1A lie on a same plane [*r.m.s* 0.0116 (2) Å] and the torsion angle N1A–N1–C7–C8 = 3.8 (2)°. The dihedral angle between this plane and its symmetry related plane (C7A/C8A/N1/N1A) is 87.67 (11)°. Each of these two middle C/C/N/N planes makes a dihedral angle of 63.81 (10)° with its adjacent benzene ring. The bond distances are of normal values (Allen *et al.*, 1987) and are comparable with a related structure (Zhao *et al.*, 2006).

In the crystal structure (Fig. 2), the molecules are arranged into zigzag chains along the *a* axis and these chains stacked along the *c* direction. The molecules are consolidated by C···Br [3.4032 (18)–3.5969 (19) Å] short contacts. C—H···π interactions were also observed (Table 1); *Cg*_1_ is the centroid of C1–C6 ring.

### Experimental

The title compound was synthesized by mixing a solution (1:2 molar ratio) of hydrazine hydrate (0.10 ml, 2 mmol) and 2-bromoacetophenone (0.54 ml, 4 mmol) in ethanol (20 ml). The resulting solution was refluxed for 5 h, yielding the white crystalline solid. The resultant solid was filtered off and washed with methanol. Colorless hexagonal-shaped single crystals of the title compound suitable for *X*-ray structure determination were recrystalized from acetone by slow evaporation of the solvent at room temperature over several days (m.p. 387–389 K).

### Refinement

H atoms were positioned geometrically and allowed to ride on their parent atoms, with *d*(C—H) = 0.93 Å for aromatic and 0.96 Å for CH_3_ atoms. The *U*_iso_ values were constrained to be 1.5*U*_eq_ of the carrier atom for methyl H atoms and 1.2*U*_eq_ for the remaining H atoms. A rotating group model was used for the methyl groups.

### Crystal data

**Table:**

C_16_H_14_Br_2_N_2_	*D*_x_ = 1.669 Mg m^−^^3^
*M**_r_* = 394.11	Melting point = 387–389 K
Orthorhombic, *P**c**c**a*	Mo *K*α radiation, λ = 0.71073 Å
Hall symbol: -P 2a 2ac	Cell parameters from 3458 reflections
*a* = 17.2162 (3) Å	θ = 3.4–35.0°
*b* = 11.8414 (3) Å	µ = 5.16 mm^−^^1^
*c* = 7.6953 (2) Å	*T* = 100 K
*V* = 1568.79 (6) Å^3^	Block, colorless
*Z* = 4	0.41 × 0.27 × 0.18 mm
*F*(000) = 776	

### Data collection

**Table:**

Bruker APEXII CCD area-detector diffractometer	3458 independent reflections
Radiation source: sealed tube	2397 reflections with *I* > 2σ(*I*)
graphite	*R*_int_ = 0.042
φ and ω scans	θ_max_ = 35.0°, θ_min_ = 3.4°
Absorption correction: multi-scan (*SADABS*; Bruker, 2005)	*h* = −27→27
*T*_min_ = 0.227, *T*_max_ = 0.462	*k* = −19→17
18529 measured reflections	*l* = −12→12

### Refinement

**Table:**

Refinement on *F*^2^	Primary atom site location: structure-invariant direct methods
Least-squares matrix: full	Secondary atom site location: difference Fourier map
*R*[*F*^2^ > 2σ(*F*^2^)] = 0.031	Hydrogen site location: inferred from neighbouring sites
*wR*(*F*^2^) = 0.081	H-atom parameters constrained
*S* = 1.02	*w* = 1/[σ^2^(*F*_o_^2^) + (0.033*P*)^2^ + 0.5992*P*] where *P* = (*F*_o_^2^ + 2*F*_c_^2^)/3
3458 reflections	(Δ/σ)_max_ = 0.001
92 parameters	Δρ_max_ = 0.75 e Å^−^^3^
0 restraints	Δρ_min_ = −0.40 e Å^−^^3^

### Special details

**Table:**

Experimental. The crystal was placed in the cold stream of an Oxford Cryosystems Cobra open-flow nitrogen cryostat (Cosier & Glazer, 1986) operating at 120.0 (1) K.
Geometry. All e.s.d.'s (except the e.s.d. in the dihedral angle between two l.s. planes) are estimated using the full covariance matrix. The cell e.s.d.'s are taken into account individually in the estimation of e.s.d.'s in distances, angles and torsion angles; correlations between e.s.d.'s in cell parameters are only used when they are defined by crystal symmetry. An approximate (isotropic) treatment of cell e.s.d.'s is used for estimating e.s.d.'s involving l.s. planes.
Refinement. Refinement of *F*^2^ against ALL reflections. The weighted *R*-factor *wR* and goodness of fit *S* are based on *F*^2^, conventional *R*-factors *R* are based on *F*, with *F* set to zero for negative *F*^2^. The threshold expression of *F*^2^ > σ(*F*^2^) is used only for calculating *R*-factors(gt) *etc*. and is not relevant to the choice of reflections for refinement. *R*-factors based on *F*^2^ are statistically about twice as large as those based on *F*, and *R*- factors based on ALL data will be even larger.

### Fractional atomic coordinates and isotropic or equivalent isotropic displacement parameters (Å^2^)

**Table:**

	*x*	*y*	*z*	*U*_iso_*/*U*_eq_	
Br1	0.634618 (11)	0.935005 (14)	0.01020 (2)	0.03110 (7)	
N1	0.53208 (8)	0.65309 (11)	0.19436 (17)	0.0228 (3)	
C1	0.64389 (9)	0.79361 (14)	−0.1054 (2)	0.0234 (3)	
C2	0.70442 (10)	0.77985 (16)	−0.2228 (2)	0.0294 (3)	
H2A	0.7383	0.8391	−0.2459	0.035*	
C3	0.71362 (10)	0.67629 (16)	−0.3054 (2)	0.0328 (4)	
H3A	0.7538	0.6662	−0.3847	0.039*	
C4	0.66330 (11)	0.58836 (16)	−0.2698 (2)	0.0312 (4)	
H4A	0.6695	0.5193	−0.3256	0.037*	
C5	0.60342 (10)	0.60310 (15)	−0.1509 (2)	0.0253 (3)	
H5A	0.5700	0.5433	−0.1274	0.030*	
C6	0.59263 (9)	0.70617 (13)	−0.06618 (19)	0.0209 (3)	
C7	0.52783 (9)	0.71762 (13)	0.06106 (19)	0.0205 (3)	
C8	0.46160 (10)	0.79632 (16)	0.0247 (2)	0.0289 (3)	
H8A	0.4138	0.7545	0.0230	0.043*	
H8B	0.4693	0.8319	−0.0860	0.043*	
H8C	0.4592	0.8529	0.1138	0.043*	

### Atomic displacement parameters (Å^2^)

**Table:**

	*U*^11^	*U*^22^	*U*^33^	*U*^12^	*U*^13^	*U*^23^
Br1	0.03424 (10)	0.01869 (9)	0.04038 (11)	−0.00163 (6)	−0.00389 (8)	0.00074 (7)
N1	0.0244 (6)	0.0181 (6)	0.0259 (6)	0.0005 (5)	0.0057 (5)	0.0004 (5)
C1	0.0236 (7)	0.0204 (7)	0.0261 (7)	0.0020 (6)	−0.0017 (6)	0.0031 (6)
C2	0.0238 (8)	0.0320 (9)	0.0324 (8)	0.0002 (7)	0.0023 (6)	0.0113 (7)
C3	0.0296 (9)	0.0395 (11)	0.0293 (8)	0.0076 (8)	0.0100 (7)	0.0063 (7)
C4	0.0337 (9)	0.0305 (9)	0.0295 (8)	0.0078 (7)	0.0072 (7)	−0.0017 (7)
C5	0.0264 (8)	0.0220 (7)	0.0275 (7)	0.0016 (6)	0.0042 (6)	−0.0004 (6)
C6	0.0216 (7)	0.0203 (7)	0.0208 (6)	0.0029 (6)	0.0005 (5)	0.0016 (5)
C7	0.0204 (7)	0.0188 (7)	0.0222 (6)	0.0006 (5)	0.0004 (5)	−0.0031 (5)
C8	0.0255 (8)	0.0368 (9)	0.0244 (7)	0.0091 (7)	−0.0007 (6)	0.0003 (6)

### Geometric parameters (Å, °)

**Table:**

Br1—C1	1.9027 (16)	C4—C5	1.389 (2)
N1—C7	1.2812 (19)	C4—H4A	0.9300
N1—N1^i^	1.398 (2)	C5—C6	1.396 (2)
C1—C2	1.389 (2)	C5—H5A	0.9300
C1—C6	1.393 (2)	C6—C7	1.491 (2)
C2—C3	1.390 (3)	C7—C8	1.499 (2)
C2—H2A	0.9300	C8—H8A	0.9600
C3—C4	1.382 (3)	C8—H8B	0.9600
C3—H3A	0.9300	C8—H8C	0.9600
			
C7—N1—N1^i^	116.45 (14)	C4—C5—H5A	119.5
C2—C1—C6	121.93 (16)	C6—C5—H5A	119.5
C2—C1—Br1	118.05 (13)	C1—C6—C5	117.65 (14)
C6—C1—Br1	119.97 (12)	C1—C6—C7	123.27 (14)
C1—C2—C3	119.09 (16)	C5—C6—C7	119.08 (14)
C1—C2—H2A	120.5	N1—C7—C6	115.40 (14)
C3—C2—H2A	120.5	N1—C7—C8	124.31 (14)
C4—C3—C2	120.19 (16)	C6—C7—C8	120.22 (13)
C4—C3—H3A	119.9	C7—C8—H8A	109.5
C2—C3—H3A	119.9	C7—C8—H8B	109.5
C3—C4—C5	120.05 (17)	H8A—C8—H8B	109.5
C3—C4—H4A	120.0	C7—C8—H8C	109.5
C5—C4—H4A	120.0	H8A—C8—H8C	109.5
C4—C5—C6	121.08 (16)	H8B—C8—H8C	109.5
			
C6—C1—C2—C3	0.8 (2)	C4—C5—C6—C1	0.1 (2)
Br1—C1—C2—C3	178.28 (13)	C4—C5—C6—C7	−179.65 (16)
C1—C2—C3—C4	−0.3 (3)	N1^i^—N1—C7—C6	−173.12 (13)
C2—C3—C4—C5	−0.2 (3)	N1^i^—N1—C7—C8	3.8 (2)
C3—C4—C5—C6	0.3 (3)	C1—C6—C7—N1	−117.18 (17)
C2—C1—C6—C5	−0.7 (2)	C5—C6—C7—N1	62.6 (2)
Br1—C1—C6—C5	−178.13 (12)	C1—C6—C7—C8	65.7 (2)
C2—C1—C6—C7	179.07 (15)	C5—C6—C7—C8	−114.47 (18)
Br1—C1—C6—C7	1.7 (2)		

Symmetry codes: (i) −*x*+1, *y*, −*z*+1/2.

### Hydrogen-bond geometry (Å, °)

**Table:**

Cg1 is the centroid of C1–C6 ring.

**Table:**

*D*—H···*A*	*D*—H	H···*A*	*D*···*A*	*D*—H···*A*
C3—H3A···Cg1^ii^	0.93	2.83	3.7246 (17)	161
C8—H8A···Cg1^iii^	0.96	2.93	3.4989 (18)	119

Symmetry codes: (ii) *x*+1, −*y*, *z*−3/2; (iii) *x*+3/2, −*y*, −*z*+3/2.
